# The Epitranscriptome and Innate Immunity

**DOI:** 10.1371/journal.pgen.1005687

**Published:** 2015-12-10

**Authors:** Mary A. O’Connell, Niamh M. Mannion, Liam P. Keegan

**Affiliations:** 1 CEITEC Masaryk University, Brno, Czech Republic; 2 Paul O’Gorman Leukaemia Research Centre, Institute of Cancer Sciences, College of Medical, Veterinary and Life Sciences, University of Glasgow, Glasgow, United Kingdom; University College London, UNITED KINGDOM

## Abstract

Our knowledge of the variety and abundances of RNA base modifications is rapidly increasing. Modified bases have critical roles in tRNAs, rRNAs, translation, splicing, RNA interference, and other RNA processes, and are now increasingly detected in all types of transcripts. Can new biological principles associated with this diversity of RNA modifications, particularly in mRNAs and long non-coding RNAs, be identified? This review will explore this question by focusing primarily on adenosine to inosine (A-to-I) RNA editing by the adenine deaminase acting on RNA (ADAR) enzymes that have been intensively studied for the past 20 years and have a wide range of effects. Over 100 million adenosine to inosine editing sites have been identified in the human transcriptome, mostly in embedded Alu sequences that form potentially innate immune-stimulating dsRNA hairpins in transcripts. Recent research has demonstrated that inosine in the epitranscriptome and ADAR1 protein establish innate immune tolerance for host dsRNA formed by endogenous sequences. Innate immune sensors that detect viral nucleic acids are among the readers of epitranscriptome RNA modifications, though this does preclude a wide range of other modification effects.

## Introduction

Conventional RNA-Seq is unable to address how much RNA modification occurs in mRNA and noncoding RNAs. Reverse transcriptases used in cDNA synthesis have evolved tolerances for diverse types of base modification in the RNA template; amazingly, they can even make cDNA copies of highly modified sections of tRNAs and rRNAs [1]. This evolutionary feature of reverse transcriptases is likely to reflect the presence of a range of modified bases in RNAs https://www.broadcastify.com/listen/feed/2822 but it also means that standard protocols for cDNA synthesis and sequence analysis do not reveal most modified bases. Until now, identifications of modified bases in mRNAs have relied mainly on mass spectrometry or antibodies specific for the modified base, or on the detection of different responses of the modified base versus the normal base to some chemical modification ([2] and references therein). However, there have been some recent developments to improve the detection of certain modifications [3].

RNA base modifications—in particular, base methylations in mRNAs and noncoding RNAs—have been described as the “epitranscriptome” [4–6], suggesting that effects of modified RNA bases also involve reader, writer, and eraser proteins. Base modification enzymes have switched between DNA and RNA substrates in evolution. Studies on adenosine to inosine (A-to-I) editing by adenine deaminases acting on RNA (ADARs) now show that innate immune nucleic acid sensors are one set of readers of modified bases in RNA [7]. It is now clear that modified bases in either DNA or RNA aid innate immune sensors in discriminating between host and viral RNAs.

## *N* ^6^-methyladenosine (m^6^A) Writers, Readers, and Erasers in mRNAs and Noncoding RNAs

The emerging roles of *N* ^6^-methyladenosine (m^6^A) have received a lot of recent attention. m^6^A does not change base-pairing preferences of RNA and cannot recode open reading frames. It has been found in approximately 7,000 mRNAs with an enrichment around the stop codon and in the 3′UTR regions of transcripts [8]. This is the RNA base modification that has been characterised in the terms of the epitranscriptome model, as the addition and removal of a methyl group is reminiscent of DNA methylation and epigenetics. The modification is introduced by IME4 in *Drosophila* and by the METTL3 and METTL14 proteins in vertebrates; YTH and hnRNP C proteins bind to RNAs containing the m^6^A base as readers, and the fat mass and obesity-associated gene (FTO) and ALKBH5 enzymes are erasers able to demethylate the m^6^A (for review, see [9]). *Drosophila Ime4* mutants fail to induce meiosis and mouse *Mettl3* mutant embryonic stem (ES) cells fail to differentiate. Pluripotency transcripts that normally have m^6^A accumulate to higher levels when they are unmodified in the *Mettl3* mutant ES cells and do not decrease to allow differentiation [10]. A possible explanation for this is that the *Mettl3* phenotype is due to an altered balance between transcript production and turnover; the reader proteins YTH and hnRNP C may facilitate the turnover of subsets of m^6^A-containing transcripts.

Although m^5^C modification at CpG islands in DNA is the canonical example of an epigenetic modification, studies on m^5^C in RNA are still at an early stage. Several different methods have been used to identify m^5^C positions in RNAs, with little overlap between these sites found within either mRNA or ncRNA (for review, [11]). The biological role of m^5^C in mRNA and ncRNA is largely unknown, and proteins binding m^5^C in RNA have not yet been identified.

## Diverse Roles of A-to-I RNA Editing by ADARs

Studies on ADAR RNA editing can also be interpreted in relation to the epitranscriptome model. The earliest work on ADARs focused on their recoding of codons in open reading frames but recent findings on ADAR1 have uncovered effects normally associated with other types of base modification.

The ADAR RNA-editing enzymes convert adenosine (A) to inosine (I) by hydrolytic deamination of adenosine bases within double-stranded (ds)RNA [12]. Individual adenosine bases are edited in pre-mRNAs when exons form short RNA hairpin structures, usually with nearby introns. Editing within exons can result in recoding of open reading frames because inosine is read as guanosine by the translational machinery [13]. Inosine prefers to form base pairs with cytosine, so ADAR RNA editing sites are easily identified since A in the genomic sequence appears as G in cDNA sequences [14]. ADARs primarily recognize duplex RNA that is in the A-form; they prefer certain bases beside the edited A but do not recognize a strong consensus sequence [15]. Studies on site-specific RNA editing have focused on ADAR2 which is enriched in the brain and is responsible for editing the Glutamine to Arginine Q/R site in the *Gria2* transcript and many of the other specific sites in CNS transcripts [16]. Editing the *Gria2* Q/R site appears to be the main function of ADAR2 as *Adar2* mutant mice, which die from seizures three weeks after birth, can be rescued by knocking-in the edited isoform *Gria2* ^*R*^ [17].

**Fig 1:**
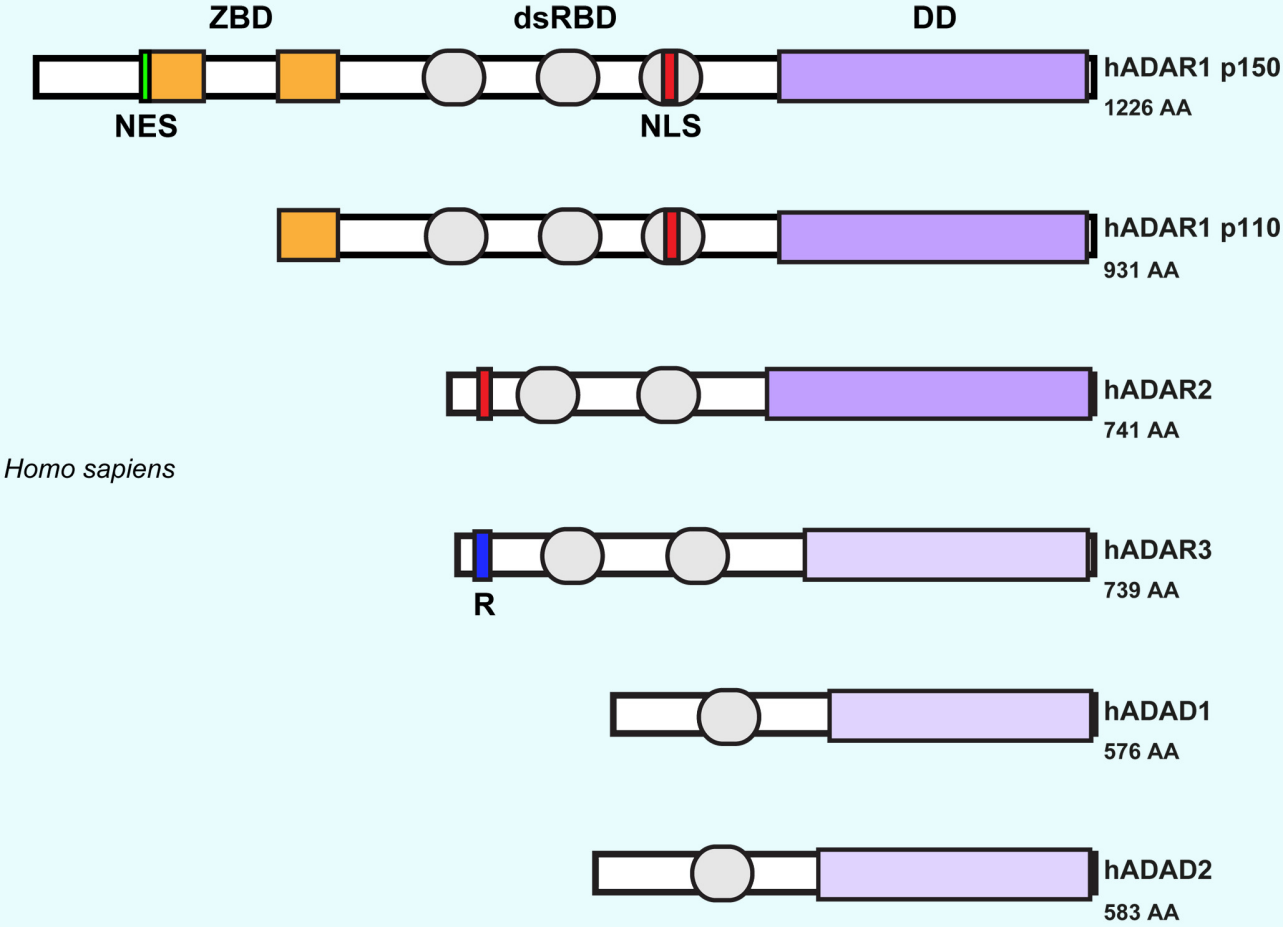
A schematic representation of the ADAR and related ADAD proteins present in humans. All proteins have dsRNA binding domains (grey box) and a C-terminal deaminase domain (purple box). A nuclear localization sequence (NLS) is marked in red, whereas the nuclear export signal (NES) present in ADAR1 is marked in green. Z-DNA binding domains are indicated by an orange box in ADAR1. A region enriched in arginine/lysine residues, R domain is present in ADAR3 (blue box). The number of amino acids is indicated on the right.

The ADAR proteins are comprised of two or three dsRNA binding domains (dsRBDs) at the amino terminus and a catalytic adenosine deaminase domain at the carboxyl terminus. Mammals have five related ADAR proteins (Fig 1). ADAR1 and ADAR2 are enzymatically active (for review, [18]) but enzymatic activity has never been demonstrated for the brain-specific ADAR3 protein, even though it closely resembles ADAR2 [19]. The more divergent testis-specific adenine deaminase domain-contain proteins (ADAD1 and ADAD2) lack key catalytic residues but are still evolutionarily conserved, presumably acting as dsRNA-binding proteins [20,21]. Conservation of deaminase domain fold structure and key active site residues show that the ADARs are members of the cytidine deaminase protein family (CDAs); different cytosine deaminases edit cytosine to uracil in RNA or DNA or both [22].

Whereas ADAR2 has a major role in site-specific editing, ADAR1 performs “promiscuous” editing of transcripts encoding repetitive elements (for review, [23]). This is reflected in their different biological roles. ADAR1 is more stable than ADAR2 and thus was the first to be identified and purified [24,25]. Unlike recombinant ADAR2, purified endogenous ADAR2 is not stable [26]. It is post-translationally regulated by phosphorylation, proline isomerization, and ubiquitination [27]. The first distinctive features of ADAR1 were its wider tissue distribution and the higher levels of expression in most tissues than ADAR2. Another distinctive feature of ADAR1 is that it expresses two different isoforms—a constitutive and predominantly nuclear 110 kDa isoform and a larger interferon-induced and mainly cytoplasmic 150 kDa isoform [28–30]. Both ADAR1 isoforms shuttle between cytoplasm and nucleus [31]. One area of controversy is whether ADAR1 is pro- or antiviral (for review, [32] and references therein). There is evidence for both points of view; however, viruses sometimes hijack the cell’s defence system so that antiviral proteins appear to have a pro-viral role [33].

Transgenic *Adar1* ^*-/-*^ mice die at day E12.5 with defects in haematopoiesis and generalized induction of interferon and the interferon-stimulated gene (ISG) transcripts [34–36]. It was presumed that the *Adar1* mutant phenotype would be due to loss of a site-specific editing event that alters a protein or microRNA required for stem cell maintenance or blood system development [36]. An intense search ensued to identify ADAR1-edited transcript(s) or noncoding RNAs. This approach proved unsuccessful and for over ten years the molecular cause of the mortality in the *Adar1* ^*-/-*^ mice remained elusive.

## Innate Immune Sensor Proteins As Readers of Inosine in dsRNA

Eventually, a different genetic approach was undertaken to rescue the *Adar1* mutant mice [7], based on the idea that the mutant defect is caused by aberrant activation of the innate immune system by cellular dsRNA and not due to a defect in site-specific editing. ADAR1 is primarily responsible for promiscuous editing of transcripts encoding repetitive elements such as short interspersed nuclear elements (SINEs) which, in humans, are Alu elements [37,38]. On average, human pre-mRNAs contain about ten embedded Alu elements. Alu elements can readily form RNA duplexes that are promiscuously edited at low levels when they lie within one or two kilobases of each other in inverted orientations; this accounts for the majority of all identified RNA editing events in human transcripts [38]. Some transcripts have Alu duplexes in their 3′ UTRs that can reach the cytoplasm [39], where dsRNA can activate the innate immune responses.

**Fig 2:**
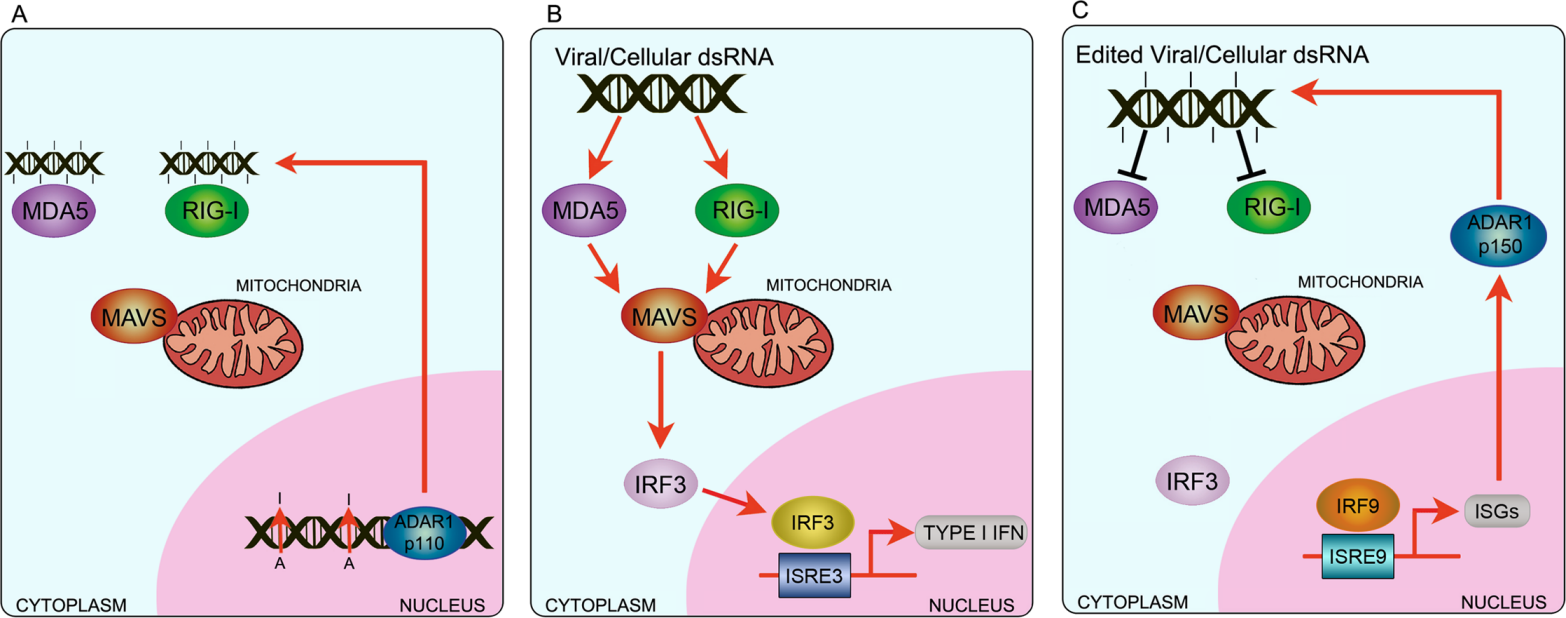
A model for the role of ADAR1 in innate immunity. (A) DsRNA that is generated during transcription is edited by ADARs. The dsRNA contain inosine that can bind to the RLRs, MDA5 and RIG-I, in the cytoplasm and inhibit their activation. (B) During a viral infection, or if ADAR1 is inactive, unedited dsRNA is present in the cytoplasm. This dsRNA binds to MDA5 and RIG-I, activating MAVS which, in turn, leads to the phosphorylation of IRF3 and its translocation into the nucleus and induces the type 1 interferon response. (C) The ADAR1 isoform p150 is induced by interferon late in the response. This isoform is predominantly cytoplasmic and it edits all dsRNA either of viral or cellular origin. This generates inosine containing dsRNA that can inhibit the RLR receptors, thus turning off the interferon response as the transcription factor IRF9 is unable to continue to transcribe the ISGs.

Viral dsRNA in the cytoplasm is detected by the innate immune sensor proteins Retinoic acid-Inducible Gene 1 (RIG-I) and Melanoma Differentiation-Associated protein 5 (MDA5) (review in [40]). These RIG-I-like (RLR), innate immune sensors bind dsRNA with RNA helicase domains; they do not unwind dsRNA, as they lack the required structure elements, but instead they scan dsRNA by using ATPase activity to translocate smoothly along it or to disassociate and re-associate [41–43]. The key adapter protein mediating antiviral responses and interferon induction by dsRNA through the RLR pathway is the mitochondrial antiviral-signalling protein (MAVS), also known as VISA, IPS-1, and CARDIF (for review, [44]). Double homozygous *Adar1*; *Mavs* mice survive to birth, and ISG transcripts that were aberrantly activated in the *Adar1* mutant embryos returned to normal in the double mutant embryos (Fig 2) [7]. Therefore, the *Adar1* mutant defect in these mice is due to an aberrant innate immune response, and ADAR1 is not required primarily for development of the embryonic haematopoietic system [34–36] nor is it essential for the function of DICER in early development [45] as, otherwise, the *Adar1*; *Mavs* mice would not survive to birth [46]. However, ADAR1 may affect these processes indirectly.

Restoring expression of either ADAR1 or an ADAR2 mutant that localizes to the cytoplasm in stably transfected *Adar1; P53* MEF cells reduces the level of transcription of ISGs, whereas catalytically inactive ADAR1 does this to an observable but considerably lower extent [7]. Thus, the *Adar1* mutant phenotype appears to be mainly due to loss of inosine in RNA, with *Mavs* rescuing as it blocks the aberrantly activated signalling from both of the RLR dsRNA sensors. The effect of inosine-containing dsRNA is dominant; dsRNA oligonucleotide-containing inosine can bind to RIGI and MDA5, thus inhibiting the innate immune response [47].

Part of the ADAR1 effect is due to an editing-independent function. A recent study demonstrated that knocking-in the *Adar1* ^*E861A*^ catalytically inactive mutation in mice gives an embryonic phenotype that is similar to, but not as severe as, the complete null [48]. The *Adar1* ^*E861A*^ catalytically inactive mutant embryos die two days later (E14.5 versus E12.5) [48], and a double mutant that eliminates just one of the RLR sensors, MDA5 (*Ifih1* ^*-/-*^), rescues the *Adar1* ^*E861A*^ mutant but not *Adar1* null mutant embryos [7]. This implies that the presence of the inactive *Adar1* ^*E861A*^ protein partially rescues the *Adar1* null mutant phenotype in the whole embryo, presumably because it still binds the most critical immune-inducing RNAs. Enzymes that introduce epigenetic modifications in chromatin also show partial rescues by catalytically-inactive mutants [49,50]. Maintaining the structure of a particular protein complex on the correct nucleic acid partially substitutes for lack of the epigenetic modification. ADARs bind to more dsRNAs than they edit, and the inosine base in a substrate does not necessarily change the binding affinity [51,52]. For epitranscriptome RNA base modifications in general, persistent binding of writer enzymes and interactions with other proteins could contribute in parallel with the base modification itself.

While it is tempting to associate the ADAR1 editing-independent activity with Alu or SINE RNAs, this remains an entirely open question. ADAR proteins are not abundant, and many adenosines in Alu RNA hairpins are edited at efficiencies below 1%. The editing-independent ADAR1 effect may involve particular immune-inducing ADAR target RNAs present in both mice and humans. ADAR1 tightly binds many of the conserved pre-mRNA structures that are site-specifically edited by ADAR2; some such RNA structures are entirely in exons or UTR regions and might reach the cytoplasm to affect innate immune sensors. The inactive ADARs, the brain specific ADAR3, and the two testis-specific ADAR-related ADAD proteins present in the genome may also bind particular RNAs that are tissue-specific (for review [23]).

The importance of epitranscriptome base modification for innate immune regulation is well illustrated by Aicardi-Goutières syndrome (AGS), which is caused by mutations in *ADAR1* [53]. AGS is a fatal childhood congenital encephalopathy with interferon overexpression, and children with ADAR1 mutations also present with childhood dystonia due to bilateral striatal neurodegeneration, again with interferon expression. One could envisage that when an AGS child with *ADAR1* mutations catches a transient viral infection, reduced ADAR1 dosage or activity leads to unedited cellular RNAs in the cytoplasm binding to RLRs, stimulating an innate immune response (Fig 2). After 12 hours, ADAR1p150, which is mainly cytoplasmic, is induced by interferon. It should edit all RNAs in the cytoplasm, thus turning off the innate immune signal to limit self-damage caused by interferon responses [7]. Resolution of the interferon response fails in the AGS patient; the inflammation is chronic and fails to correctly resolve.

## Innate Immune Sensors Read Other Epitranscriptome Modifications

Data from the Weissman group has demonstrated that in vitro transcribed RNA containing various modified nucleotides such as m^5^C, m^6^A, m^5^U, pseudouridine, or 2′-O-methylated nucleotides dampen the innate immune response when transfected into mammalian dendritic cells, whereas RNA that is unmodified will stimulate it [54–56]. The responses observed are likely to involve RLR as well as TLR sensors.

In the case of inosine effects on RLRs, the inosine–uracil (I-U) wobble base pair weakens dsRNA base pairing and several I-U base pairs together cause dsRNA melting [57]. It is likely that it is this perturbation of the helical structure by the I-U wobble base pair that is detected by RLRs [7]. However, many other types of modified bases could also perturb dsRNA structure sufficiently for innate immune sensors to distinguish self dsRNA from the perfect dsRNA that is formed directly from virus replication. For instance, m^6^A also weakens base pairing in dsRNA even though it does not alter base pairing preferences [58]. 2′-O-methylated groups in the minor groove would also be easy for RLRs to detect, and these are known to prevent innate immune induction by dsRNA.

Levels of base modifications often change in response to stress or virus infection. Pseudouridine has been found in mRNA in both yeast and mammalian cells in the past year by three groups [59–61], all using a similar method of treating the RNA with CMC (*N*-cyclohexyl-*N′*-(2-morpholinoethyl)-carbodiimide metho-p-toluenesulphonate) a chemical that forms a stable interaction with pseudouridine [62]. When this RNA is subsequently used for cDNA synthesis, reverse transcriptase will terminate when it encounters the artificial base derivative. When a stress is applied to cells, such as heat shock of the yeast cells or serum starvation of the mammalian cells, this generally increases the level of pseudouridine; however, the biological function of this stress-induced increase in pseudouridine in mRNA is unclear [63].

Base modification is important in the conflict between virus and host because viruses can benefit from evading host innate immune responses if they can capture some modifications. Thus, m^6^A occurs in some viral RNA at a higher level than would be predicted from a random occurrence; for example, Rous sarcoma viral mRNA has seven m^6^A sites, whereas SV40 has more than ten [64]: it has been shown that TLR 3 is not activated when m^6^A is present in RNA [56].

Helm and co-workers have demonstrated that stimulation of the innate immune receptor TLR7, by tRNA purified from *Escherichia coli* but not by tRNA from human cells in PBMCs [65], is due to at least three different modifications on the human tRNAs—two of which are methylations of ribose 2′hydroxyls on tRNA guanosines 18 and 34. These naturally occurring modifications in native tRNA not only prevent stimulation of TLR7 by the modified tRNA but also appear to antagonize TLR7 signalling to suppress immunostimulation by unmodified test tRNAs, similar to the inhibitory effect of inosine in dsRNA on the activation of RLRs.

Conflicts between self and non-self nucleic acids are much older than the animal innate immune systems [66], and tRNA modifications may also affect these conflicts. For instance, the γ-toxin from *Kluyveromyces lactis* cleaves tRNAs^Glu/Gln/Lys^ from *Saccharomyces cerevisiae* on the 3′ side of an anticodon base that is modified. The γ-toxin recognizes the cleavage sites due to the 5-methoxycarbonylmethyl-2-thiouridine wobble base modification present on these tRNAs in *S*. *cerevisiae* but not in *K*. *lactis* [67–69].

## Recent Advances

Some modified bases lead to particular patterns of misincorporation at the modified base position in standard RNA-Seq and indications of RNA modification events can be obtained from existing RNA sequence databases [70]. New RNA-Seq protocols identify modified sites by cloning cDNAs synthesised using lower nucleotide concentrations and more discriminating reverse transcriptases [71] that give increased misincorporation and termination. Also, more detailed examinations of modified base effects on RLRs are beginning to be investigated [72]. However, considering the abundance and variety of modification in RNA, it will take the development of new technology before one can be confident that the encoded RNA is truly being sequenced.

## Conclusion

As Albert Einstein once said, “Fundamental ideas of science are essentially simple.” The use of host RNA modification to distinguish between host and parasite nucleic acids is reminiscent of the restriction and modification that occurs in bacterial DNA. With the plethora of modifications in RNA, it’s easy to envisage these would generate a unique bar code that would be species-specific, allowing intricate “self”-“non-self” discrimination.
